# A flavoprotein supports cell wall properties in the necrotrophic fungus *Alternaria brassicicola*

**DOI:** 10.1186/s40694-016-0029-3

**Published:** 2017-01-06

**Authors:** Sandrine Pigné, Agata Zykwinska, Etienne Janod, Stéphane Cuenot, Mohammed Kerkoud, Roxane Raulo, Nelly Bataillé-Simoneau, Muriel Marchi, Anthony Kwasiborski, Guillaume N’Guyen, Guillaume Mabilleau, Philippe Simoneau, Thomas Guillemette

**Affiliations:** 1 IRHS, Agrocampus-Ouest, INRA, Université d’Angers, SFR 4207 QuaSaV, 49071 Beaucouzé, France; 2 UMR 6502, Institut des Matériaux Jean Rouxel, 2, Rue de la Houssinière, BP 32229, 44322 Nantes Cedex 3, France; 3 Present Address: Laboratoire Ecosystèmes Microbiens et Molécules Marines pour les Biotechnologies, IFREMER, Rue de l’île d’Yeu, BP 21105, 44311 Nantes Cedex 3, France; 4 Plateforme SCIAM, Institut de Biologie en Santé, CHU, Université d’Angers, 4, Rue Larrey, 49933 Angers Cedex, France

**Keywords:** Monooxygenase, Cell wall, Melanin, Fungus, Flavoprotein

## Abstract

**Background:**

Flavin-dependent monooxygenases are involved in key biological processes as they catalyze a wide variety of chemo-, regio- and enantioselective oxygenation reactions. Flavoprotein monooxygenases are frequently encountered in micro-organisms, most of which require further functional and biocatalytic assessment. Here we investigated the function of the *AbMak1* gene, which encodes a group A flavin monooxygenase in the plant pathogenic fungus *Alternaria brassicicola*, by generating a deficient mutant and examining its phenotype.

**Results:**

Functional analysis indicates that the AbMak1 protein is involved in cell wall biogenesis and influences the melanization process. We documented a significant decrease in melanin content in the Δ*abmak1* strain compared to the wild-type and complemented strains. We investigated the cell wall morphology and physical properties in the wild-type and transformants using electron and atomic force microscopy. These approaches confirmed the aberrant morphology of the conidial wall structure in the Δ*abmak1* strain which had an impact on hydrophilic adhesion and conidial surface stiffness. However, there was no significant impairment in growth, conidia formation, pathogenicity or susceptibility to various environmental stresses in the Δ*abmak1* strain.

**Conclusion:**

This study sheds new light on the function of a fungal flavin-dependent monooxygenase, which plays an important role in melanization.

**Electronic supplementary material:**

The online version of this article (doi:10.1186/s40694-016-0029-3) contains supplementary material, which is available to authorized users.

## Background

Flavin-dependent monooxygenases are involved in a wide variety of biological processes, such as biosynthesis, catabolism and detoxification of various natural compounds and xenobiotics, in both prokaryotes and eukaryotes. They catalyze the incorporation of one atom of molecular oxygen into the substrate and these oxygenation reactions include, for instance, hydroxylation, epoxidation, Baeyer–Villiger oxidation or sulfoxidation (for reviews, see [1–3]). Specific monooxygenase-driven transformations are usually hard to achieve without using these enzymatic catalysts, which is why such enzymes (particularly flavin-dependent monooxygenases and cytochrome P450 monooxygenases) are of great interest for synthetic purposes. An analysis of genome sequences revealed that flavoprotein monooxygenases are frequently encountered in micro-organisms, most of which require functional and biocatalytic assessments [2, 3]. Eight groups of flavin monooxygenases can be distinguished on the basis of their structural features and functions [2]. Group A flavin monooxygenases are single-component enzymes that contain typical FAD binding regions and rely on NAD(P)H as their external electron donor. Typical class A substrates are aromatic compounds containing an hydroxyl or amino group. Only about 70 group A monooxygenase members are currently known, many of which do not yet have an Enzyme Commission (EC) number.

In phytopathogenic fungi, one of the best known group A flavin monooxygenases is the MAK1 protein from *Nectria haematococca*. MAK1 specifically hydroxylates medicarpin and maackiain, converting them into less fungitoxic derivatives [4]. Medicarpin and maackiain are antifungal phytoalexins produced by many legumes, and are thought to be important components of the defense response of these legumes to certain fungal pathogens. In a previous study, in *Alternaria brassicicola*, we identified a gene encoding a class A flavin monooxygenase which was found to be upregulated by camalexin, the major phytoalexin produced by *Arabidopsis thaliana* [5]. *A. brassicicola* causes black spot disease in a wide range of Brassicaceae plants and is routinely used as a model necrotrophic pathogen in studies with *A. thaliana*. In the present study, we investigated the function of the *AbMak1* gene by generating a knockout mutant and examining its phenotype. Unexpectedly, our functional analyses showed that this protein is involved in cell wall biogenesis and influences the melanization process. Like other filamentous fungi, *Alternaria* species synthesize melanin via a 1,8-dihydroxynaphthalene (DHN) intermediate [6]. Melanins constitute a group of related pigments that are polymers of phenolic compounds, although the exact arrangement of these phenolic subunits is generally unclear [7]. These ubiquitous pigments are known to provide protection against damaging effects of environmental stresses such as ultraviolet (UV) irradiation, enzymatic lysis, extreme temperatures, oxidizing agents and ionizing radiation [8]. In addition, they play a role in the pathogenesis of some human and plant pathogenic fungi [9, 10].

## Results

### AbMak1 encodes a class A flavoprotein monooxygenase

**Fig. 1:**
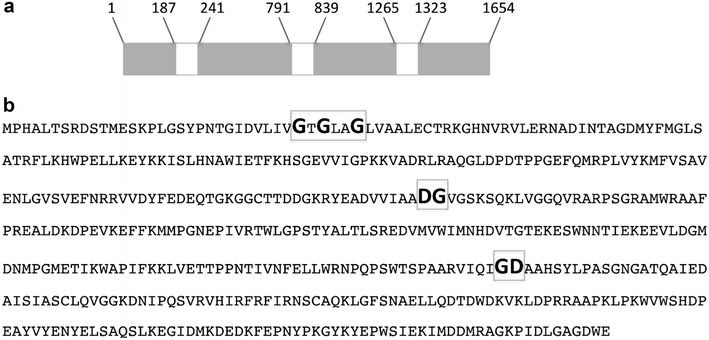
Sequence features of the *AbMak1* gene and corresponding protein. **a** Schematic *AbMak1* gene structure. The predicted intronic sequences are indicated in *white*. **b** Amino acid sequence of AbMak1. Typical motifs of flavin monooxygenases belonging to group A are indicated in *bold*

The camalexin-induced sequence P1B3 (GenBank accession No. DY543080) was previously identified in *A. brassicicola* as an EST encoding a predicted protein having matches with the flavin-containing monooxygenase MAK1 described in *N. haematococca* [5]. The corresponding protein, here referred to as AbMak1 and annotated AB02358.1 through the interactive JGI fungal portal MycoCosm (http://genome.jgi.doe.gov/Altbr1/Altbr1.home.html), displayed strong sequence similarities with hypothetical proteins from other Dothideomycetes species. For instance, the resulting AbMak1 protein had 95% identity or more to the corresponding proteins described in *Pyrenophora teres* or in various *Cochliobolus* species. AbMak1 also displayed high similarities with homologs from other lineages, such as *Podospora anserina* (76%) and *Aspergillus niger* (40%). A lower degree of identity (26%) was obtained by alignment with the *N. haematococca* MAK1 protein. Only one copy of the gene was present in the draft genome sequence of *A. brassicicola*. Automatic annotation at the locus encoding AB02358.1 predicted four introns and an encoded 528 amino acid protein. Nevertheless, examination of the transcribed sequence showed that automated prediction of the fourth intron was wrong (data not shown), resulting in a protein containing 497 amino acids (Fig. 1; Additional file 1: Figure S1). This protein contains typical motifs of flavin monooxygenases belonging to group A. The first fingerprint sequence is the Rossmann fold, or *βαβ*-fold (containing the GXGXXG sequence), which is crucial for binding the ADP moiety of FAD [11]. The second FAD binding motif contains the GD sequence which contacts the riboflavin moiety of FAD [12]. The last fingerprint contains a highly conserved DG motif, which is involved in binding the pyrophosphate moieties of both FAD and NADPH [13].

### Generation of the AbMak1 disruption mutant and major morphological traits

**Fig. 2:**
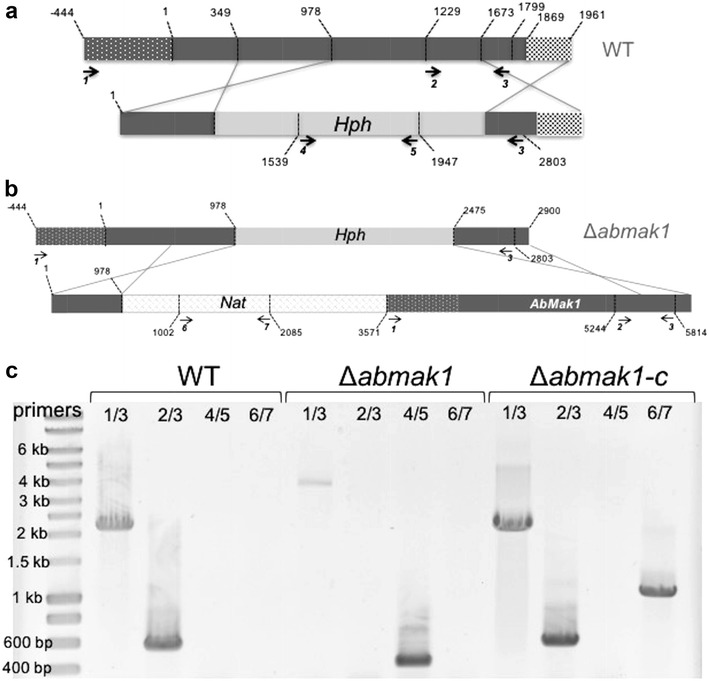
Generation of Δ*abmak1* and Δ*abmak1*-*c* by homologous recombination. **a** Schematic representation of the *AbMak1* locus (*grey box*) in the wild-type and the replacement construct with the Hyg B resistance cassette (*Hph* gene) and flanking sequences. **b** Schematic representation of the *AbMak1* locus in the Δ*abmak1* mutant and the replacement construct with the nourseothricin resistance cassette (*Nat* gene) and flanking sequences. *Arrows* indicate the position of primers used for PCR screening of mutants. **c** Gel electrophoresis of PCR products obtained from template DNA of the wild-type, Δ*abmak1* or Δ*abmak1*-*c* strains with the indicated primer pairs. Molecular sizes (kb) were estimated based on a 1 kb ladder (lane L, Eurogentec, Seraing, Belgium). Primer 1: CACAGCAACCTTGAACACGA; primer 2: CATTCCTCAATCTGTCCGCG; primer 3: TGGTCGTTACACCAGGGATC; primer 4: GGCGAAGAATCTCGTGCTTT; primer 5: CATCACAGTTTGCCAGTGATAC; primer 6: GTTGTAAAACGACGGCCAGT; primer 7: GGCTTCGTGGTCATCTCGTA

Disruption of *AbMak1* in *A. brassicicola* was accomplished by replacing a part of the *AbMak1* ORF with a hygromycin B (Hyg B) resistance cassette (Fig. 2). One Hyg B-resistant transformant was generated (Δ*abmak1*) after transformation of protoplasts of the wild-type strain, regeneration and purification by single-spore isolation. PCR screening, carried out using primers homologous to the hygromycin resistance cassette and genomic sequence outside of the flanking regions, confirmed that integration of the replacement construct occurred by homologous recombination at the targeted loci for this mutant (Fig. 2). Disruption of the coding region in the transformant was further confirmed using internal primers. One complemented strain (Δ*abmak1*-*c)* was obtained by reintroducing the wild-type copy of *AbMak1* into the Δ*abMak1* mutant. In this case, the replacement cassette included a 629 bp 5′ region of the gene *AbMak1*, the *Nat* gene cassette conferring resistance to nourseothricin, and the wild-type allele of the *AbMak1* gene with its native promoter. The homologous recombination event occured at the previously transformed locus in the Δ*abMak1* mutant (as shown in Fig. 2) and led to the replacement of the the *Hph* gene cassette by the *Nat* gene cassette and to the intoduction of a wild-type copy of the *AbMak1* gene.

**Fig. 3:**
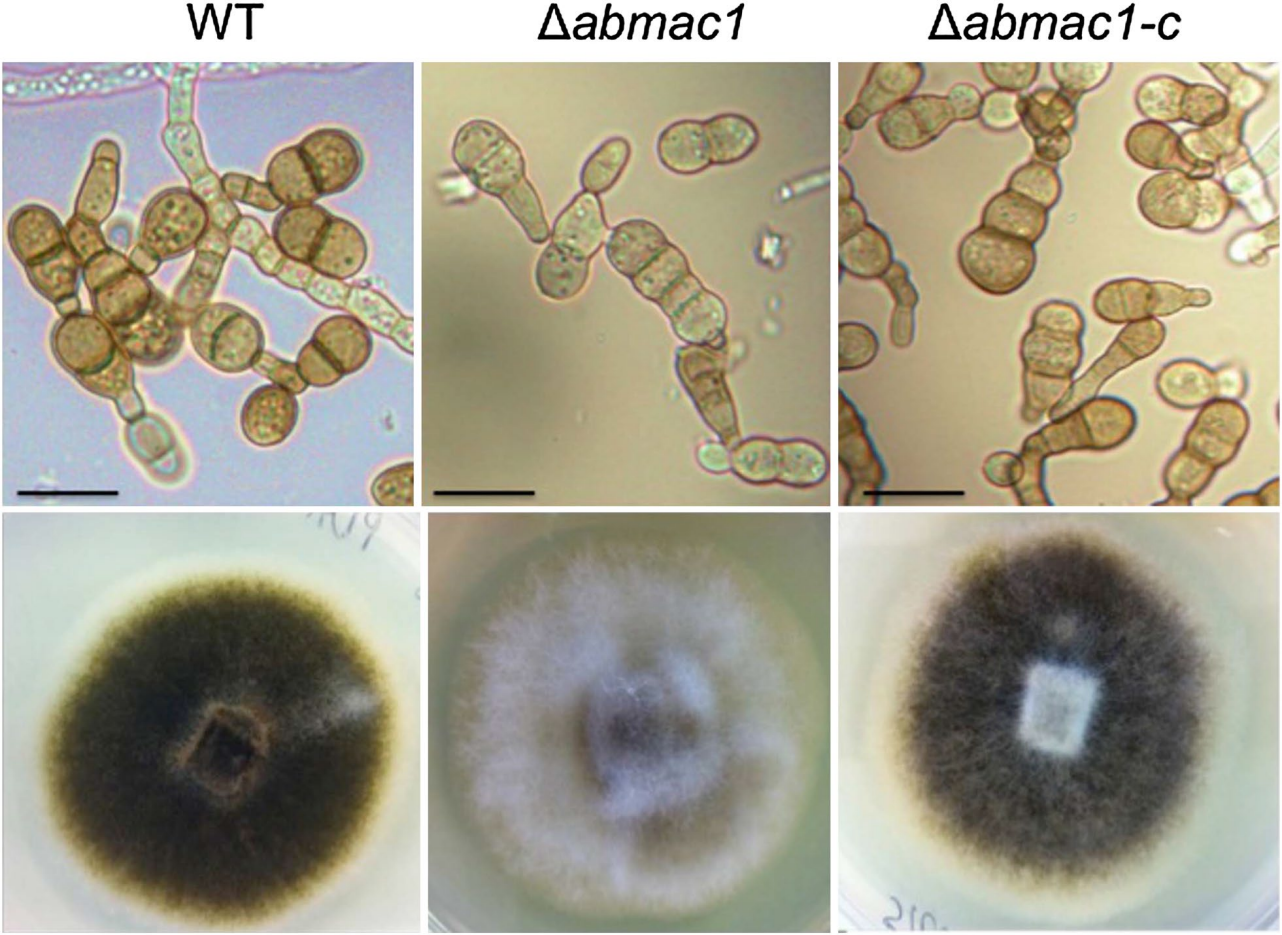
Morphological features of *A. brassicicola* wild-type, Δ*abmak1 and* Δ*abmak1*-*c* strains. *Top panels* show representative microscopic images of conidia. *Scale bars* indicate 20 μm. *Bottom panels* show representative macroscopic images of a colony

**Fig. 4:**
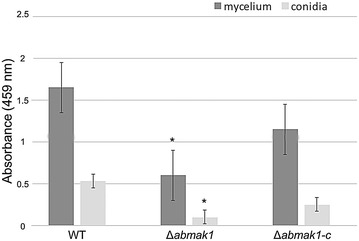
Semi-quantitative assay of melanin based on spectrophotometry readings at 459 nm on extracted melanin from either isolated conidia or mycelium. Mycelia or isolated conidia from 7-day-old cultures were extracted by an alkali–acid method to obtain melanin. The amount of melanin was extrapolated from the photometry absorbance results at 459 nm, as previously reported by Babitskaia et al. [14] and Alviano et al. [48]. *Asterisk* indicate a significant difference between the mutant and the parental isolate (Student’s *t* test, P < 0.01)

Microscopic observations revealed that Δ*abmak1* and Δ*abmak1*-*c* mutants displayed a normal hyphal morphology, growth rate and conidiation rate on standard potato dextrose agar (PDA) medium. The most obvious morphological change was the color of Δ*abmak1* conidia that appeared lighter than that of conidia produced by the reference strain or the Δ*abmak1*-*c* strain (Fig. 3). In order to validate a potential pigmentation alteration in Δ*abmak1* conidia, a semi-quantitative assay of melanin, based on spectrophotometry readings at 459 nm on melanin extracted from either isolated conidia or mycelium, was first used, as described by Babitskaia et al. [14]. The results showed a significant decrease in the melanin content of the Δ*abmak1* sample compared to the wild-type sample and partial recovery of the melanin level in the Δ*abmak1*-*c* strain (Fig. 4).

Another method, based on calculation of the integral intensity of electron paramagnetic resonance (EPR) spectra, was used and confirmed this significant melanin loss in *∆abmak1* cell walls. Different kinds of melanin biopolymers, such as eumelanin, pheomelanin and neuromelanin, exist and they have some interesting features such as redox properties [15]. These properties are due to delocalization of an electron between orthoquinone and catecholic moieties, giving rise to semiquinone free radicals. These radicals offer the opportunity for melanins to be involved in one- and two-electron redox reactions. The semiquinone free radicals trapped in melanin are responsible for the paramagnetic properties of melanin detectable by EPR. More precisely, melanin contains both o-semiquinone free radicals upon which unpaired electrons are localized on oxygen atoms with spin of ½ and biradicals with spin of 1 [15, 16]. EPR spectroscopy detects the absorption of energy relative to the transition of unpaired electrons from a low to a higher energy level. EPR spectra provide information about the concentration of paramagnetic centers, their type and distribution (homogeneous or non-homogeneous) in the samples [16].

**Fig. 5:**
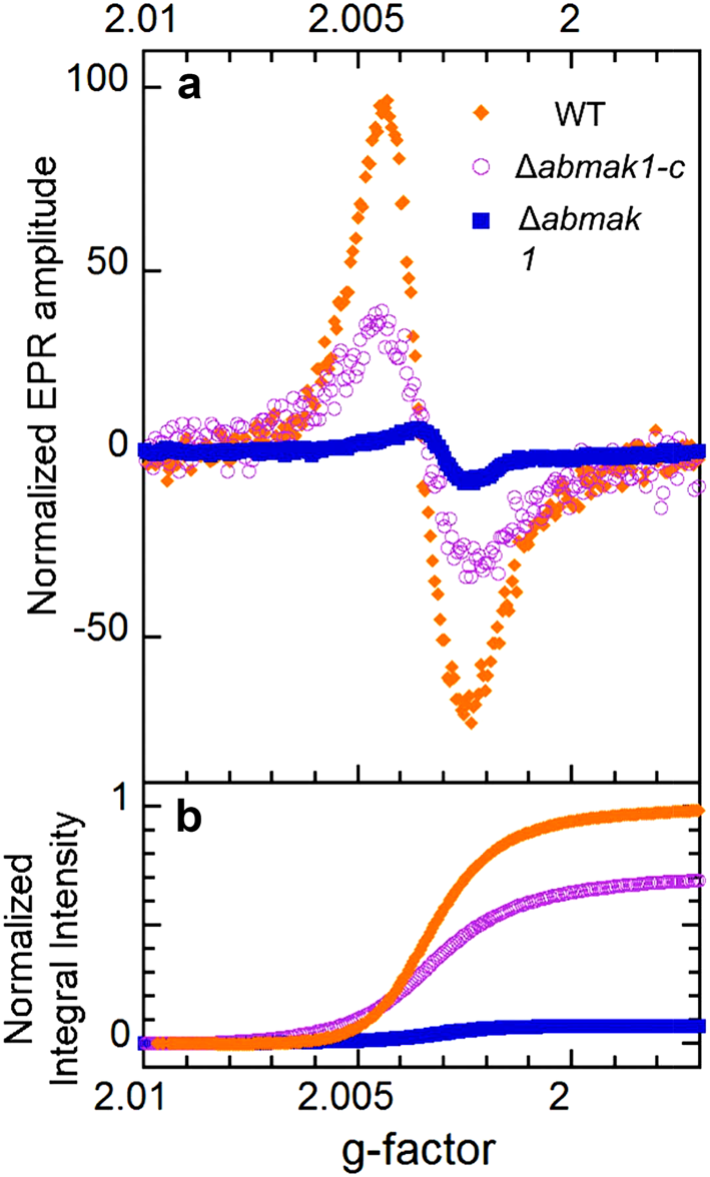
Calculation of the integral intensity of electron paramagnetic resonance (EPR) spectra. **a** Normalized X-band EPR spectra of *A. brassicicola* wild-type, Δ*abmak1* Δ*abmak1*-*c* samples represented as a function of the g-factor. **b** Normalized integral intensities with respect to the maximum intensity of the wild-type

Figure 5a shows three spectra recorded in the linear regime, with microwave powers lower than typical P_1/2_ values of 1 mW. Such low P_1/2_ values are in good agreement with those previously reported by Sarna and Hyde [17] for different melanin samples. These spectra were normalized with respect to both the experimental conditions and sample mass. For all samples, the signal shape was in general slightly asymmetric, resembling that expected from a powder pattern arising from a π–electron radical with an approximately axial g-tensor [17]. The same S = ½ free-radical signal was observed for the three samples, with a g-factor of 2.0035 for the wild-type and *∆abmak1*-*c* and of 2.0030 for *∆abmak1*, respectively. These very close values—consistent with those reported in the literature for o-semiquinone free radicals—indicated that the same type of melanin was present in the three samples [15, 16]. Indeed, the weak difference of 5e−4 in the g-factor is much smaller than the dispersion of g-values published for melanin, i.e. in the 2.0030–2.0060 range [15, 16]. The integral intensity of EPR spectra was calculated to estimate the relative contents of free radicals in the three samples. Indeed, the free radical concentration is directly proportional to this value [16]. The integral intensity calculation was done without any assumption on the spectra shape and the area under the absorption curves was obtained by double integrations. Figure 5b presents the integral intensities normalized with respect to that of the wild-type. These results clearly showed that the relative free radical contents markedly decreased in *∆abmak1* relative to the wild-type and *∆abmak1*-*c*. Indeed, the normalized intensities were about 7 ± 2 and 70 ± 10% for *∆abmak1* and *∆abmak1*-*c*, respectively. Hence, these results suggest a substantial loss of melanin in *∆abmak1* and partial recovery of melanin in *∆abmak1*-*c.*

### Conidial wall ultrastructure imaged by electron microscopy

**Fig. 6:**
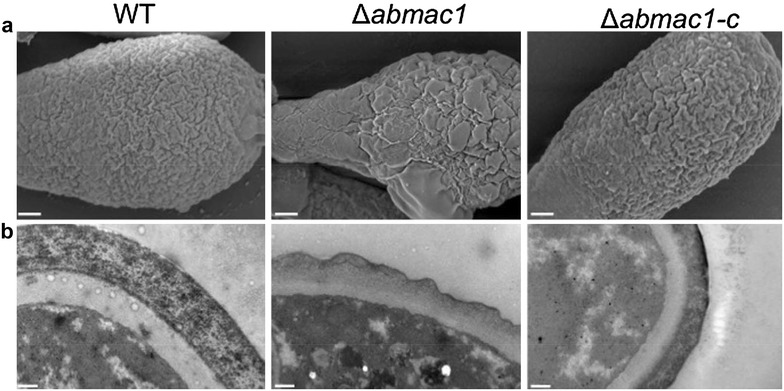
Ultrastructure of the conidial walls. **a** Scanning electron micrographs of 7-day-old wild-type, Δ*abmak1 and* Δ*abmak1*-*c* conidia (*scale bars* 1 μm). **b** Transmission electron micrographs of 7-day-old wild-type, Δ*abmak1* and Δ*abmak1*-*c* conidia (*scale bars* 0.2 μm)

We investigated the morphology of the wild-type and transformant conidia using scanning (SEM) and transmission (TEM) electron microscopy. SEM images of the conidial surface showed typical ornamentation for the wild-type and *∆abmak1*-*c* strains (Fig. 6a). However, the *∆abmak1* mutant displayed a highly altered conidial surface. Ornamentation was not found to be as regular as in the wild strain and surface subsidences were clearly visible.

The aberrant morphology of the mutant conidia was confirmed on TEM images of the conidial wall ultrastructure (Fig. 6b). Typical conidial walls are composed of several superimposed layers, with a thick electron transparent inner layer, a middle cell wall layer and a thin electron-dense outermost layer. TEM showed that, in the conidial cell wall of *∆abmak1*, the contours of the outermost layer appeared irregular and that the separation between primary and secondary walls was no longer visible. It is also apparent that the thickness of *∆abmak1* walls (511 nm ± 120) is greatly reduced compared to the thickness of *∆abmak1*-*c* (687 nm ± 113) and wild-type walls (809 nm ± 128) (Student’s *t* test, P < 0.01). The *∆abmak1*-*c* cell wall ultrastucture was found to be much closer to that of wild-type cell walls.

### Investigation of the conidial surface by atomic force microscopy (AFM): imaging and force spectroscopy measurements

The AFM images of *A. brassicicola* conidia presented in the Additional file 2: Figure S2A revealed the presence of ornamentation on the wild-type cell wall surface, in agreement with the SEM observations (Fig. 6). In contrast, the *∆abmak1* conidial surface was significantly affected in the *∆abmak1* mutant, which led to less regular ornamentation and the presence of large smooth zones on the conidial surface. The cell wall surface morphology of *∆abmak1*-*c* conidia showed more similarities to that of the wild-type than to that of *∆abmak1*. However, neither the wild-type nor its mutants presented specific nanoscale structures on the cell wall surface, which remained perfectly smooth (Additional file 2: Figure S2B).

**Fig. 7:**
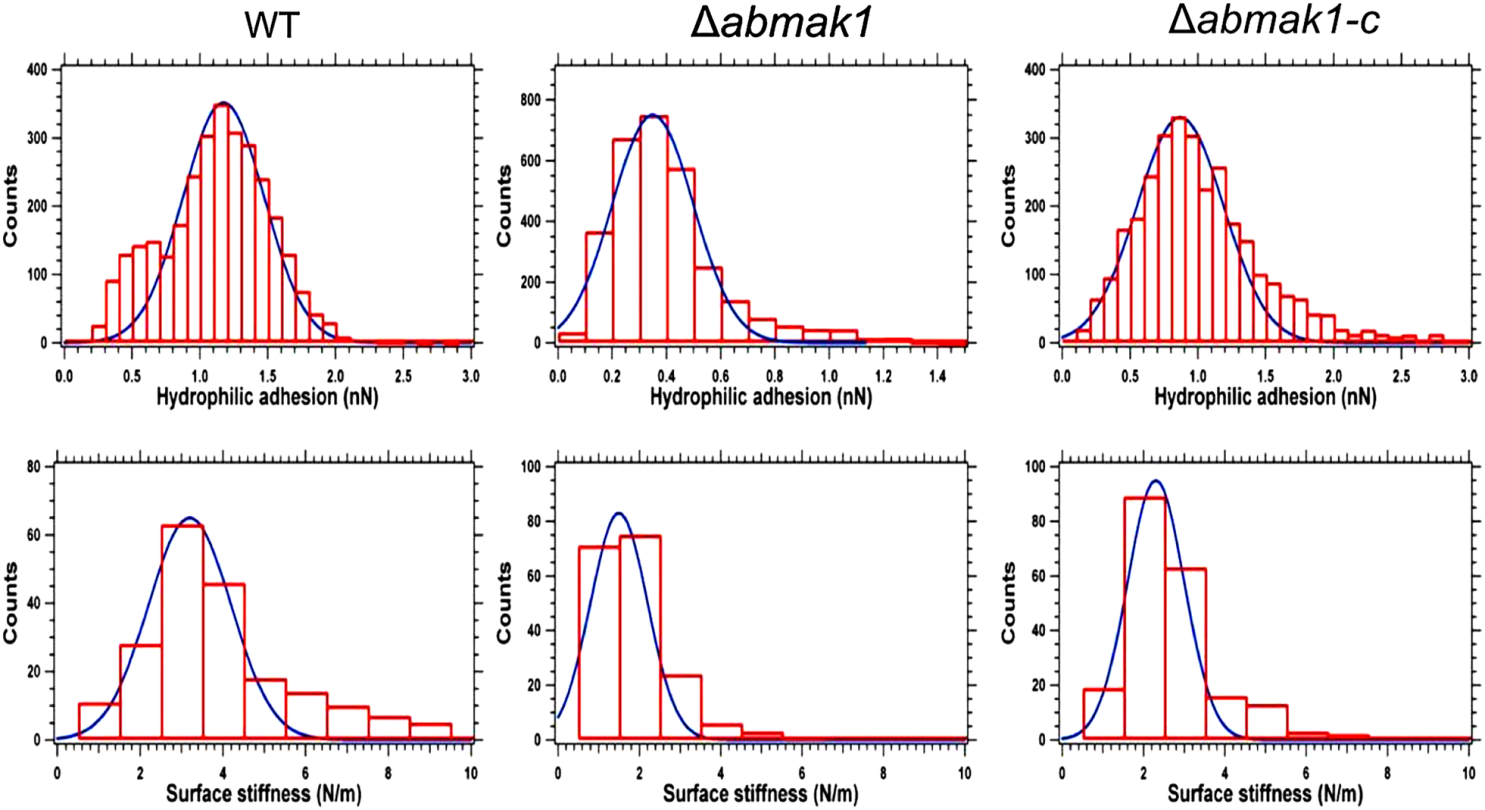
Histograms of OH/OH hydrophilic adhesion forces and local surface stiffness measured on **a** the wild-type, **b** *∆abmak1* and **c** *∆abmak1*-*c* strains

To investigate the chemical nature of the cell wall surface and probe any difference between the cell wall composition of Abra43 and that of *∆abmak1*, non-specific force-curves were measured with OH-modified tips (Fig. 7). Chemical force spectroscopy measurements using OH-modified probes revealed the presence of hydrophilic components, such as polysaccharides, OH groups of melanin and glycoproteins, on the outer conidial cell wall surface [18]. Force-curves recorded on the surface of wild-type conidia with OH tips showed large adhesion forces of 1.15 ± 0.3 nN, whereas a threefold lower value of 0.35 ± 0.15 nN was obtained on the *∆abmak1* conidial surface. Hydrophilic OH/OH adhesion forces of 0.9 ± 0.3 nN measured on the *∆abmak1*-*c* conidial surface were very close to those obtained for the wild-type. The uncertainties, which correspond to the experimental dispersion, are determined from the Gaussian fits performed on each histogram (Fig. 7). A decrease in OH/OH adhesion measured on the *∆abmak1* conidial surface suggested that some cell wall components bearing OH groups were lacking on its surface. On the contrary, similar OH/OH adhesion values obtained on the wild-type and the *∆abmak1*-*c* cell wall surfaces indicated that their composition was similar.

Measurement of the conidial surface stiffness of different samples seemed to confirm that the conidial cell wall surface composition differed between them (Fig. 7). Indeed, twofold lower cell wall stiffness of 1.5 ± 0.5 N m^−1^ was measured on the *∆abmak1* surface in comparison to the stiffness values of 3.2 ± 1 and 2.5 ± 0.7 N m^−1^ obtained on the wild-type and the *∆abmak1*-*c* conidial cell wall surfaces, respectively. As for hydrophilic adhesion measurements, the uncertainties are obtained from the Gaussian fits of the stiffness measurements, and they reflect the experimental dispersion. The significantly lower elasticity measured on the *∆abmak1* surface highlighted that the mutation strongly impacted the cell wall composition. The hydrophilic adhesion and conidial surface stiffness results obtained for *∆abmak1*-*c* tended to be closer to those of the wild-type than to those of *∆abmak1*.

### Susceptibility of the ∆abmak1 mutant to stress conditions

Monitoring growth in solid PDA medium or in liquid PDB medium did not reveal any significant effect of *AbMak1* disruption, compared to the parental and complemented strains, on the mycelium growth rate, conidia germination and initial hyphal growth (data not shown). The results of analyses of growth curves in liquid medium supplemented with H_2_O_2_, allyl-isothiocyanate (Al-ITC) and camalexin or in solid PDA medium supplemented with Congo red (CR) and Calcofluor white (CFW) were used to assess the susceptibility of the monooxygenase mutant to oxidative stress, plant defense metabolites and cell wall stress. As shown in Table 1, none of the strains (Δ*abmak1* and Δ*abmak1*-*c)* showed increased susceptibility to any of these stresses compared to the wild-type. As PDA is a relatively rich medium, we also monitored fungal growth in a minimal medium (10 g L^−1^ glucose, 1.65 g L^−1^ (NH_4_)2SO_4_, 15 g L^−1^ agar, 1 g L^−1^ KH_2_PO_4_, 0.5 g L^−1^ KCl, 0.5 g L^−1^ MgSO_4_, 7H_2_O, 0.01 g L^−1^ FeSO_4_, 7H_2_O). We did not noticed any significative susceptibility to plant defense metabolites and cell wall stress in this particular growth condition (data not shown).

**Table 1:** Susceptibility of *A. brassicicola* wild-type, Δ*abmak1 and* Δ*abmak1*-*c* strains to different stress conditions

	H_2_O_2_		ITC	Camalexin	CR		CFW	
	5 mM	10 mM	2.5 mM	25 μM	200 mg L^−1^	400 mg L^−1^	200 mg L^−1^	400 mg L^−1^
WT	24 ± 4	84 ± 14	18 ± 5	51 ± 4	42 ± 7	55 ± 5	62 ± 5	73 ± 5
*Δabmak1*	33 ± 5	97 ± 1	20 ± 5	45.5 ± 2	46 ± 2	59 ± 5	61 ± 5	76 ± 3
*Δabmak1*-*c*	42 ± 5	89.5 ± 8	27 ± 8	51.5 ± 8	42 ± 2	56 ± 7	58 ± 5	81 ± 8

The results are expressed as the percentage of inhibition in treated samples compared to the control without additive. Conidia of each genotype were used to inoculate microplate wells containing standard PDB medium supplemented with the appropriate compound. Nephelometric growth was automatically recorded for 33 h at 24 °C. Each condition was tested in triplicate and the experiments were repeated twice. The areas under the curves were used to calculate the percentages of inhibition for each treatment compared to the control growth curves. Values are means of three biological repetitions and represent the percentage growth inhibition under stress conditions compared with standard growth conditions

### Pathogenic behavior of replacement mutants on vegetative organs

*Brassica oleracea* leaves were inoculated with drops of conidia suspension (10^5^, 10^4^ or 10^3^ conidia/mL) to test the effects of targeted *AbMak1* gene knockout on pathogenicity (Additional file 3: Figure S3). The wild-type, *∆abmak1* and *∆abmak1*-*c* were all able to produce typical symptoms and, as determined from the lesion sizes at various inoculum loads, no significant decreases in aggressiveness were recorded for the mutants. Regardless of the inoculated strain, small necrotic symptoms were already observed at 3 days post-inoculation (dpi) and they continued to expand into large typical necrotic areas surrounded by chlorotic halos at 6 dpi. During late stages of infection, necrotic spots exhibited a dense conidia formation on the surface.

**Fig. 8:**
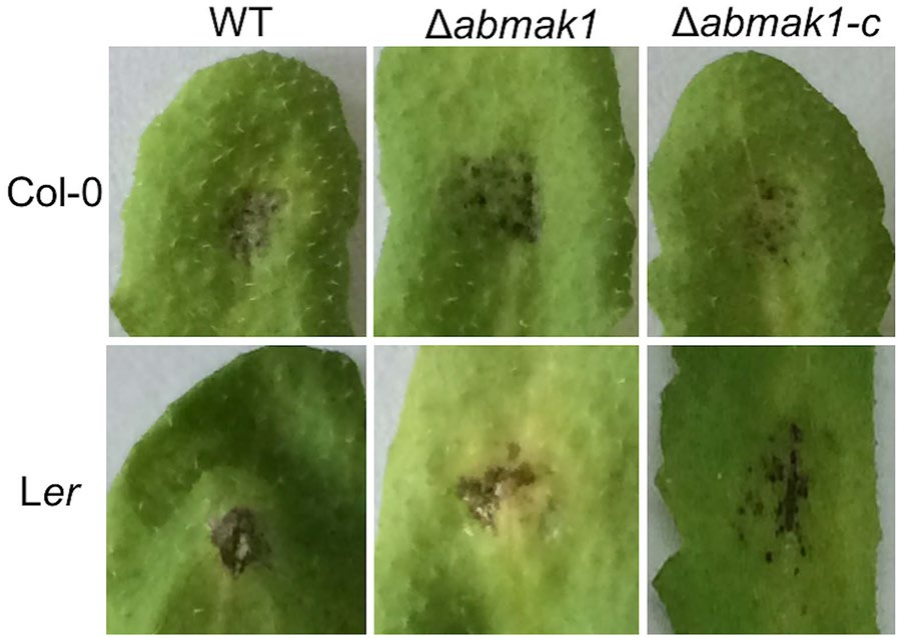
Representative symptoms obtained by inoculation of wild-type, Δ*abmak1* and Δ*abmak1*-*c* on Columbia (Col-0) and Landsberg erecta (L*er*) *A. thaliana* ecotypes

Inoculation experiments were also performed on leaves of two *A. thaliana* ecotypes: Landsberg erecta (L*er*) and Columbia (Col-0). The wild-type and mutants were all able to produce typical symptoms and visual evaluation of the necrotic lesion area at 6 dpi showed no alteration in mutant aggressiveness (Fig. 8).

## Discussion

We investigated the role of AbMak1, a group A flavin monooxygenase, in the plant pathogenic fungus *A. brassicicola* by generating a disruption mutant for the corresponding gene. Group A flavin monooxygenases comprise single-component enzymes that combine flavin reduction and monooxygenation in one polypeptide chain. They use FAD as a prosthetic group and mainly NADPH as an electron donor [19]. Well studied examples of enzymes belonging to subclass A are p-hydroxybenzoate hydroxylase, which is involved in degradation of aromatic compounds, and squalene monooxygenase, which catalyzes the first oxygenation step in sterol biosynthesis [20, 21]. Based on sequence homology and the gene expression profile, we initially hypothesized that AbMak1 could be involved in the metabolization of plant phytoalexins, as previously reported for the flavoprotein protein MAK1 from the filamentous fungus *Nectria haematococca*. Indeed, MAK1 is known to specifically hydroxylate the legume phytoalexins medicarpin and maackiain, converting them to less fungitoxic derivatives [4]. Moreover, this hypothesis was consistent with the fact that *AbMak1* was overexpressed upon exposure to camalexin, the major phytoalexin in *A. thaliana* [5]. However, a major contradictory result is that the mutant was not affected in its susceptibility to camalexin (Table 1) or to other cruciferous phytoalexins (data not shown). More generally, there was no significant impairment in growth, conidia formation or pathogenicity of the *∆abmak1* mutant. These results showed that AbMak1 was not a MAK1 ortholog in *A. brassicicola*.

As an unexpected phenotype, we observed that the loss of function of AbMak1 altered the melanin content. Like other dematiaceous fungi, *Alternaria* species produce 1,8-dihydroxynaphthalene (1,8-DHN) melanin that accumulates mainly in conidia cell walls. This ubiquitous pigment protects them from the damaging effects of environmental stress, contributes to the ability of fungi to survive in harsh environments and allows overwintering or dormancy of fungal propagules [22, 23], while also playing a role in fungal pathogenesis. In human pathogens, fungal melanin can modulate the host immune response by interfering with the normal function of phagocytic cells or altering the cytokine levels [24, 25]. Melanin is also critical to host invasion in some plant pathogens, such as *Magnaporthe oryzae*, by providing mechanical strength to the appressoria and allowing the organisms to penetrate plant tissues [26]. *A. brassicicola* does not use this mechanical strategy to penetrate host tissues, which may explain why the pathogenicity of the *∆abmak1* strain is not affected. As an *AbMak1* homologous gene exists in *M. oryzae*, it would be interesting to determine the extent to which these genes are involved in its pathogenicity. The lack of effect of melanin deficiency on the pathogenicity has also been reported in other plant-pathogenic fungi [27, 28]. Cho et al. [6] reported the functions of Amr1, a transcription factor that regulates melanin biosynthesis in *A. brassicicola*. These authors determined that *Δamr1* mutants were melanin-deficient but, unexpectedly, more virulent than the wild-type, suggesting that loss of the gene was beneficial to pathogenesis. RNA-seq analysis of interactions during late-stage pathogenesis revealed that the expression of a subset of genes involved in the melanin biosynthesis pathway was regulated by Amr1. AB02358.1, that encodes AbMak1, belongs to this subset. In contrast, many hydrolytic enzyme-coding genes were expressed at higher levels in Amr1 mutants than in the wild-type during pathogenesis, indicating that this subset of genes was negatively regulated by the transcription factor during this infection process. The authors speculated that this transcription factor promotes long-term survival due to its role in melanin biosynthesis, at the expense of virulence, thus contributing to the success of *A. brassicicola* as a competitive saprophyte and plant parasite. Interestingly, four other genes located in the vicinity of *AbMak1* were also found to be regulated by Amr1 (Additional file 4: Figure S4) suggesting that this region represents a gene cluster involved in melanin biosynthesis. One gene (AB02359.1) encode a cytochrome P450 and the other three (AB02355.1, AB02356.1 and AB02357.1) encode hypothetical proteins. Deletion of these genes should be considered and could lead to obtain more marked phenotypes.

The physical conidia surface properties were also investigated in the different *A. brassicicola* strains by atomic force microscopy. Chemical force spectroscopy measurements using OH-modified probes revealed a potential increase in *∆abmak1* conidial surface hydrophobicity since some cell wall components bearing OH groups are lacking on its surface. It should be noted that typical hydrophobic rodlet layers, which have been visualised by AFM examination of the *A. fumigatus* conidial surface [29], have not been observed in the *A. brassicicola* wild-type or in *A. brassicicola* mutants. These rodlet layers are formed by the self-assembly of particular amphiphatic proteins called hydrophobins, that are able to coat hydrophobic or hydrophilic surfaces and reverse their hydropathy character [30]. According to our observations, it therefore seems that these proteins do not participate in the hydropathy profile of *A. brassicicola* conidia and they are not altered by *AbMak1* disruption. The *∆abmak1* surface also exhibited a significantly lower elasticity compared to the wild-type or complemented strain. This character is probably linked to the decreased thickness of *∆abmak1* walls we observed on TEM images. This result highlighted the fact that the mutation not only impacted the surface hydropathy character but also, more generally, the cell wall structure and properties. However, this alteration did not seem to contribute to the marked loss of conidia adherence properties since the pathogenicity on leaves was not impaired. Contrary to our results, the complete loss of melanin in *A. fumigatus* led to the lack of some hydrophobic components on the conidial surface and modified the conidial adhesion to laminin and fibronectin [29]. Another mechanism by which pigment may contribute to virulence concerns its ability to confer some resistance to reactive oxygen species (ROS), a major host antimicrobial effector system. Jahn et al. [31] reported that an *A. fumigatus* isolate lacking conidial pigmentation displayed higher susceptibility to oxidative attack in vitro. In this study, the *AbMak1* mutant did not show increased susceptibility to hydrogen peroxyde or allyl-ITC, which are known to induce intracellular ROS accumulation [32]. More generally, it should be noted that the marked phenotypes, such as the increased susceptibility to oxidants or the loss of virulence that were reported in other studies on fungal melanins, were obtained from nonpigmented isolates. As shown in Fig. 4, the *A. brassicicola AbMak1* mutant did not exhibit a complete melanin defect. This partial melanin deficiency observed in Δ*abmak1* could therefore explain the lack of increased susceptibility to the applied stresses.

At present, it is hard to determine how the monooxygenase AbMak1 specifically acts on the melanin structure. Melanin is an amorphous polymer of phenolic compounds that is both hydrophobic and negatively charged [33]. Nevertheless, the precise physicochemical nature of melanin is not yet fully understood [34], mainly because melanin is insoluble, so many traditional biochemical techniques are unsuitable for studying this pigment [7, 9]. However, microscopic studies have revealed that melanins form granular particles localized in cell walls, where they are likely crosslinked to polysaccharides [35–37]. The wide variety of pathogenic dematiceous fungi synthesize their melanins from 1,8-dihydroxynaphthalene (DHN)-melanin from the precursor molecules acetyl coA or malonyl coA. The first step is the synthesis of 1,3,6,8-tetrahydroxynaphthalene (1,3,6,8-THN), which is catalyzed by a polyketide synthase. Then, a series of reduction and dehydration reactions produce the intermediates scytalone, 1,3,8-trihydroxynaphthalene, vermelone, and finally 1,8-dihydroxynaphthalene (DHN), whose polymerization leads to melanin formation [7, 9]. We do not believe that the AbMak1 protein is involved in the early stages of synthesis since these steps have been relatively well studied in fungi [9, 38], and also because the application of pyroquilon, an inhibitor of the hydroxynaphtalene reductase, did not modify the Δ*abmak1* phenotype compared to the wild-type (data not shown). Most of the later reaction steps involved in melanin synthesis would require further investigation, and we favor the hypothesis that the protein is involved in melanin polymerization or in crosslinking to cell wall components, such as chitin [39], via a hydroxylation step.

Detailed insight into the DHN-melanin synthesis process in fungi is important primarily because this pigment contributes to virulence in several human and plant pathogenic fungi. Enzymes involved in the DHN-melanin biosynthetic pathway are thus emerging targets for the development of selective fungicides since this pigment is not synthesized in host organisms [40]. Biological control strategies have also been envisaged to limit the accumulation and persistence of plant pathogens by degrading their melanin content or inhibiting its production [41].

## Conclusions

In this study, we identified a fungal flavin-dependent monooxygenase that plays a role in DHN-melanization. Mutation of the gene encoding AbMak1 resulted in major alteration of the cell wall structure, including a decrease in the melanin content and a probable modification of its chemical structure. The physical and chemical properties of the conidia surface were also altered but not enough to impact the pathogenicity and susceptibility of the fungus to various stress conditions. As homologous genes are present in other Ascomycota genomes, this enzyme likely has a major role throughout this phylum. As the definition of the melanin structure is beyond our current technological capability, the exact impact of this flavin-dependent monooxygenase on the melanin chemical structure remains unclear.

## Methods

### Strains and growth conditions

The *A. brassicicola* wild-type strain Abra43 used in this study has previously been described [42]. For routine cultures, fungi were maintained at 24 °C by transferring hyphal plugs on 3.9% (w/v) PDA (Difco) or on agar-solidified Vogel’s medium N supplemented with 1.2% (wt/vol) sucrose. For radial growth assays, agar disks were cut from the margin of a 7-day-old colony growing on PDA and were transferred onto the centre of PDA medium supplemented with the compounds under investigation (at concentrations specified in the Results) and incubated at 24 °C. Colony diameters were measured daily and used for calculation of radial growth (mm day^−1^). To study hyphal growth in liquid media, conidial suspensions (10^5^ spores mL^−1^, final concentration) were inoculated into microplate wells containing the appropriate test substances in PDB in a total volume of 300 µL. Microplates were placed in a laser-based microplate nephelometer (NEPHELOstar, BMG Labtech) and growth was monitored automatically over a 30 h period, as described by Joubert et al. [43]. Data were exported from Nephelostar Galaxy software in ASCII format and further processed in Microsoft Excel.

### Generation of the targeted gene disruption mutant

The gene disruption cassettes were generated using the double-joint PCR procedure described by Yu et al. [44]. The selectable marker inserted in the PCR constructs corresponded to the *Hph* gene cassette (1436 bp) from pCB1636 [45] or the *Nat* gene cassette (2150 bp) from pNR [46] conferring resistance to hygromycin B and nourseothricin, respectively. The final products of each disruption construct consisted of the chosen selective marker with 0.5–1.0 kb 5′ and 3′ spart of the targeted gene as illustrated in Fig. 2. These products were purified and used to transform *A. brassicicola* protoplasts as described in [47]. The Hyg B resistant mutants were selected and prescreened by PCR with relevant primer combinations to confirm integration of the replacement cassette at the targeted locus. The gene replacement mutants were further purified by three rounds of single-spore isolation. The *A. brassicicola* wild-type Abra43 was used to obtain the single hygromycin resistant transformant strain *∆abmak1*. The *∆abmak1* genotype was used to obtain the complemented nourseothricin resistant *∆abmak1*-*c* strain.

### Melanin extraction

After 7 days of culture on PDA medium, the mycelium (entire colony) or only the conidia were harvested and used for melanin pigment extraction. Melanin was extracted as previously reported by Babitskaia et al. [14] and Alviano et al. [48]. Briefly, samples were lyophilized and, for each genotype, the same amounts of powder were incubated in 2% NaOH (dilution coefficient 1:10) at 100 °C for 2 h in a water bath. The extract was cooled and acidified with concentrated HCl to pH 2.0. The coagulated pigment was separated by centrifugation at 6000*g* for 15 min and dissolved in 0.1 M HCl. Finally it was dialysed against distilled water and lyophilized. The amount of melanin was determined from the photometry absorbance results at 459 nm.

### Infection assays

*Arabidopsis thaliana* plants were grown to the 8- to 12-leaf stage in controlled environment rooms (21–19 °C day and night temperature respectively) and a 8 h light photoperiod. *Brassica oleracea* plants were grown in a greenhouse for 5 weeks. For inoculations, 5 μL drops of *A. brassicicola* conidia suspension (10^5^, 10^4^ or 10^3^ conidia/mL in water) were deposited on intact leaves from 5 week-old plants. Drops of sterile water were applied on control plants. The plants were then maintained under saturating humidity (100% relative humidity) in a plastic box. Symptoms were observed at 6 dpi.

### Electron microscopy

The conidial wall ultrastructure was investigated by TEM and SEM using conidial suspensions obtained from 7-day-old cultures on PDA. Concerning the TEM sample preparation, successive steps of fixation, post-fixation, dehydratation and embedding in Epon were carried out as previously described [49]. Thin sections were contrasted with uranyle acetate and lead citrate and examined under a JEM-2010 transmission electron microscope (Jeol, Paris, France). SEM samples were prepared as described in [49]. After drying by the critical-point method, specimens were then sputtercoated with a thin carbon layer and examined under a JEOL JSM 6301-F scanning electron microscope (Jeol, Paris, France).

### AFM imaging and surface property measurement

The surface of *A. brassicicola* conidia was imaged using a NanoWizard^®^ atomic force microscope (JPK Instruments AG, Berlin, Germany) operating in intermittent contact mode under ambient conditions. For imaging, a standard rectangular cantilever (Nanosensors NCL-W) was used at a free resonance frequency of 165 kHz and a typical spring constant of about 40 N m^−1^. The tip radius curvature was ~10 nm. For adhesion measurements, gold-coated cantilevers (Olympus, Hambourg, Germany) with a spring constant of 0.01 N m^−1^ were functionalized by immersion in 1 mM 11-mercapto-1-undecanol (Sigma-Aldrich) solution in ethanol for 14 h before rinsing with ethanol. Using these functionalized cantilevers, hydrophilic adhesion force measurements were performed on the conidial surface in ultrapure water [18]. A detailed analysis of the force-distance curves was performed using JPK Data Processing software (JPK Instruments AG). From these curves (2048 measurements), the mean hydrophilic adhesion was extracted from Gaussian fits performed on the histograms. For elasticity measurements, silicon nitride cantilevers having a calibrated spring constant of 0.05 N m^−1^ (Cantilevers MSCT, Veeco) were used in contact mode under ambient conditions. From the force-distance curve measurements, JPK Data Processing software was used to fit the linear part of the approach curves and then to estimate the local surface stiffness [50].

### EPR measurement

Melanin samples obtained from an entire 7 day-old colony were examined at room temperature with a Bruker-Elexsys X-band (9.78 GHz) electron paramagnetic resonance (EPR) spectrometer using a magnetic modulation field at 100 kHz. For the EPR measurements, the three samples were located in thin walled glass tubes with an external diameter of 3 mm. Masses of all samples were determined. The EPR spectra were recorded at different microwave powers in the 0.04–40 mW range. The P_1/2_ experimental parameter, which is the incident microwave power at which the signal is half as great as it would be in the absence of microwave power saturation, was estimated to avoid microwave saturation of the spectral line.

## Additional files

**Additional file 1: Figure S1.** Nucleotide sequence of *AbMak1*. The predicted intronic sequences are indicated in bold.

**Additional file 2: Figure S2.** AFM amplitude images of *A. brassicicola* strains. (**A**) Conidia of the wild-type (6 μm x 5 μm), *Δabmak1* (8 µm x 6 µm) and *Δabmak1*-*c* (10 µm x 12 µm) and (**B**) conidial surface (1.5 µm x 1.5 µm).

**Additional file 3: Figure S3.**Pathogenic behaviour of *A. brassicicola* wild-type, Δ*abmak1* and Δ*abmak1*-*c* strains. *B. oleracea* leaves were inoculated with 5 μL drops of conidia suspensions (10^5^, 10^4^ or 10^3^ conidia/mL in water). Transformants were inoculated on the right part of the central vein and compared with the parental strain inoculated on the left part of the same leaf. Symptoms were measured at 6 dpi. Values are means of three biological repetitions.

**Additional file 4: Figure S4.** Schematic map of the genomic region including *AbMak1* and flanking genes. This map was generated and modified from the Alternaria Genomes Database (http://alternaria.vbi.vt.edu/index.html). Genes are indicated as red boxes and positions of introns by white bars.
