# Supplementary material for: A flavoprotein supports cell wall properties in the necrotrophic fungus Alternaria brassicicola
Source: Fungal Biol Biotechnol. 2017 Jan 6;4:1. doi: 10.1186/s40694-016-0029-3 (PMC5611651; doi:10.1186/s40694-016-0029-3)
Supplement: Supplementary file 1 — Additional file 1: Figure S1. Nucleotide sequence of AbMak1. The predicted intronic sequences are indicated in bold. [file 40694_2016_29_MOESM1_ESM.pdf]

ATGCCTCACGCTCTTACTTCCCGCGACTCCACGATGGAGTCGAAGCCACTGGGCTCGTACCC
CAACACTGGCATCGACGTCCTCATCGTGGGTACTGGTCTGGCTGGTCTGGTCGCTGCCCTCG
AATGCACACGCAAGGGACACAATGTTCGTGTACTGGAGAGGAACGCAGACATCAACACAGCT
G**GTGAGCAGCATCCTCACACTACAGTCTTGACGCATTGCTCATAATGCAATTTAG**GTGACAT
GTACTTCATGGGACTCAGTGCGACCCGCTTCTTGAAGCACTGGCCTGAACTGCTCAAGGAGT
ACAAGAAGATATCTCTTCACAATGCCTGGATCGAGACCTTTAAGCACTCTGGCGAGGTTGTC
ATCGGACCCAAGAAAGTAGCAGACCGCCTCCGTGCACAAGGGCTGGACCCTGATACTCCTCC
TGGCGAGTTCCAGATGCGCCCGCTCGTCTACAAGATGTTTGTTAGTGCCGTAGAGAACCTCG
GTGTTTCTGTAGAGTTCAATCGTAGGGTTGTAGACTACTTCGAGGACGAGCAGACTGGCAAG
GGTGGCTGCACCACCGACGACGGCAAGCGCTACGAGGCAGATGTAGTCATCGCGGCCGATGG
CGTTGGGTCGAAGAGCCAGAAGCTTGTTGGCGGACAAGTGCGCGCGAGACCCTCTGGAAGGG
CCATGTGGCGTGCTGCTTTCCCCAGAGAGGCTCTTGACAAGGACCCGGAGGTCAAGGAGTTC
TTCAAGATGATGCCAGGCAATGAGCCAATCGTCAGGACATGGCTGGG**GTGAGCTTTTTGTAC**
GTGCACAGGTCGAAGCTTTACTAACGCTCGCAGCCCCTCCACATACGCCCTCACTCTGTCTC
GCGAGGATGTCATGGTTTGGATTATGAATCACGACGTCACCGGTACTGAGAAGGAGTCGTGG
AACAACACCATCGAGAAGGAAGAGGTTCTTGACGGCATGGACAATATGCCTGGTATGGAGAC
CATCAAGTGGGCGCCCATCTTCAAGAAGCTTGTCGAGACCACCCCACCCAACACCATTGTTA
ACTTTGAGCTCCTCTGGCGCAACCCCCAACCTAGCTGGACCTCTCCAGCGGCCCGCGTCATC
CAGATCGGTGATGCTGCACACTCATATCTACCAGCTTCTGGTAATGGTGCGACCCAGGCCAT
CGAGGATGCCATCTCGATAGCCTCCTGTCTGCAGGTTGGCGGCAAGGACAACATTCCTCAAT
CTGTCCGCGTACACATTCGCTTCCG**GTAAGCTAATATCCAACATTCACGCCCGCTACATCCA**
CATGCTAACACGACATGATAGCTTTATCCGTAACTCCTGCGCCCAAAAGCTCGGTTTCTCCA
ACGCTGAACTTCTCCAAGACACAGACTGGGACAAGGTCAAGCTTGACCCTCGCCGTGCCGCT
CCCAAGCTGCCCAAGTGGGTATGGTCTCACGACCCTGAGGCCTACGTATACGAGAACTATGA
GCTGAGTGCGCAGTCACTCAAGGAAGGCATTGACATGAAGGATGAAGACAAGTTCGAGCCCA
ACTACCCCAAGGGCTACAAGTATGAGCCTTGGAGCATCGAGAAGATCATGGATGACATGAGG
GCGGGTAAGCCGATTGACCTTGGAGCTGGTGACTGGGAG***TAA***
