# Supplementary figures and images for: A flavoprotein supports cell wall properties in the necrotrophic fungus Alternaria brassicicola
Source: Fungal Biol Biotechnol. 2017 Jan 6;4:1. doi: 10.1186/s40694-016-0029-3 (PMC5611651; doi:10.1186/s40694-016-0029-3)

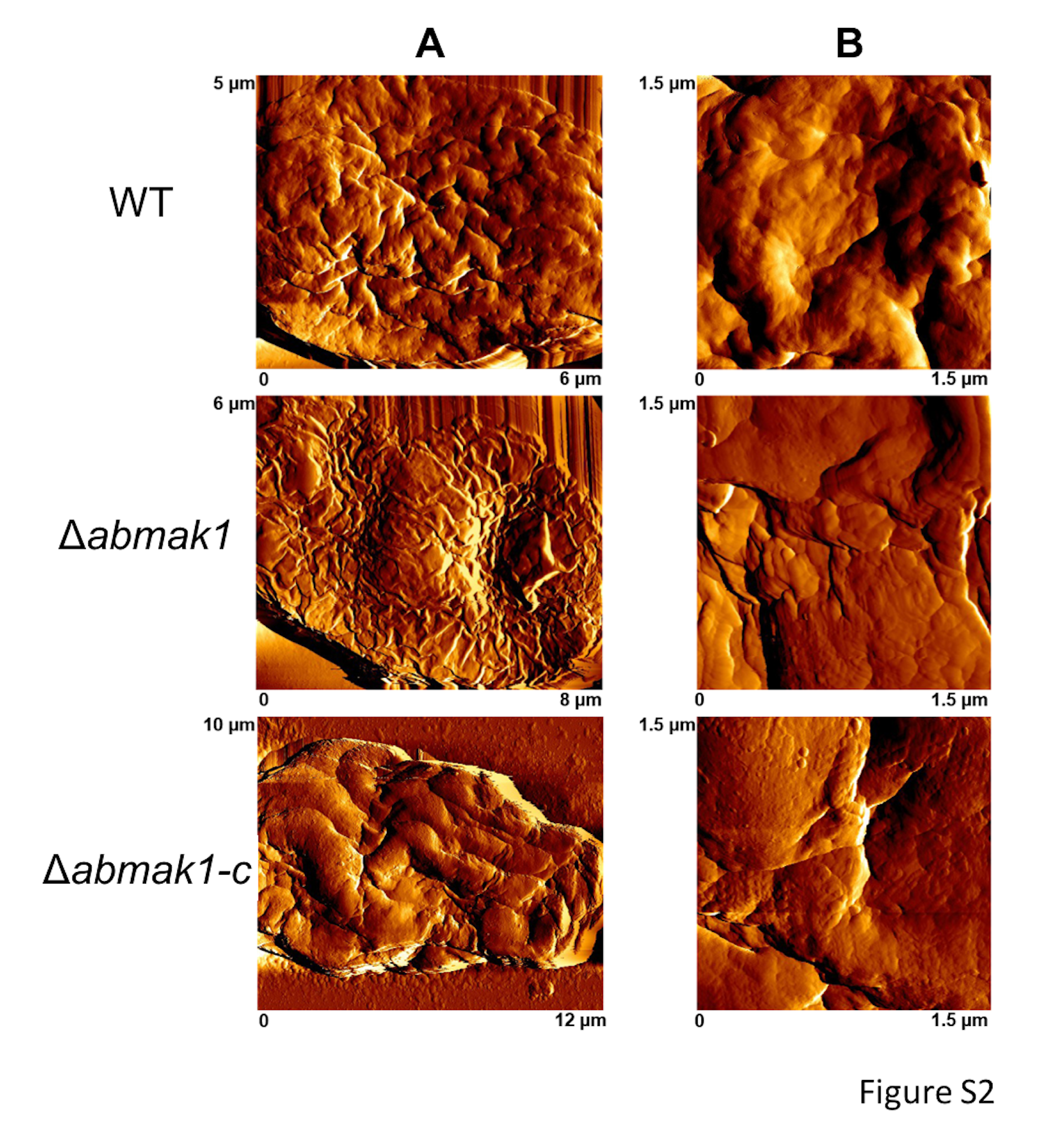

Supplement: Supplementary file 2 — Additional file 2: Figure S2. AFM amplitude images of A. brassicicola strains. (A) Conidia of the wild-type (6 μm x 5 μm), Δabmak1 (8 µm x 6 µm) and Δabmak1-c (10 µm x 12 µm) and (B) conidial surface (1.5 µm x 1.5 µm). [file 40694_2016_29_MOESM2_ESM.tiff]

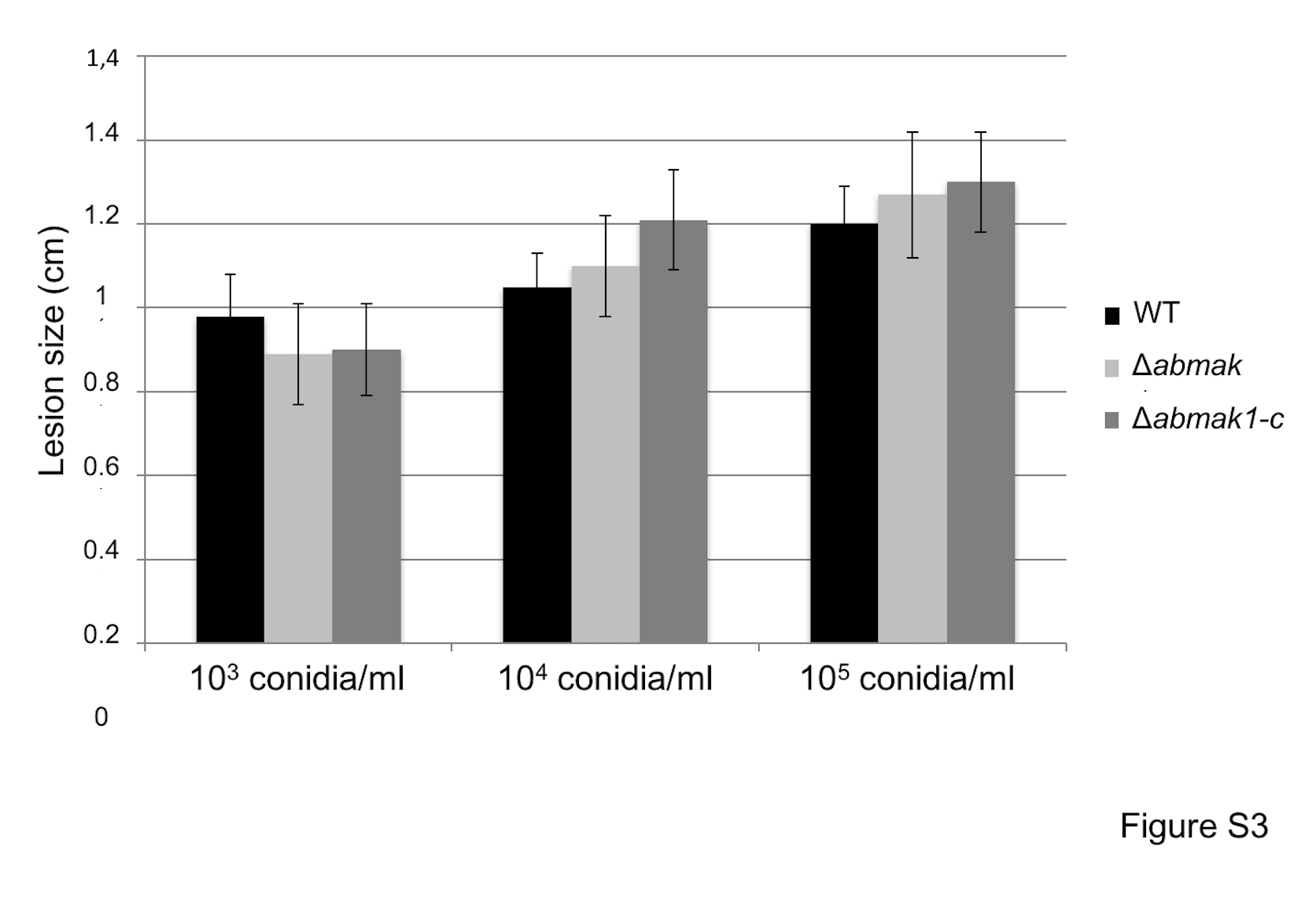

Supplement: Supplementary file 3 — Additional file 3: Figure S3.Pathogenic behaviour of A. brassicicola wild-type, Δabmak1 and Δabmak1-c strains. B. oleracea leaves were inoculated with 5 μL drops of conidia suspensions (105, 104 or 103 conidia/mL in water). Transformants were inoculated on the right part of the central vein and compared with the parental strain inoculated on the left part of the same leaf. Symptoms were measured at 6 dpi. Values are means of three biological repetitions. [file 40694_2016_29_MOESM3_ESM.png]

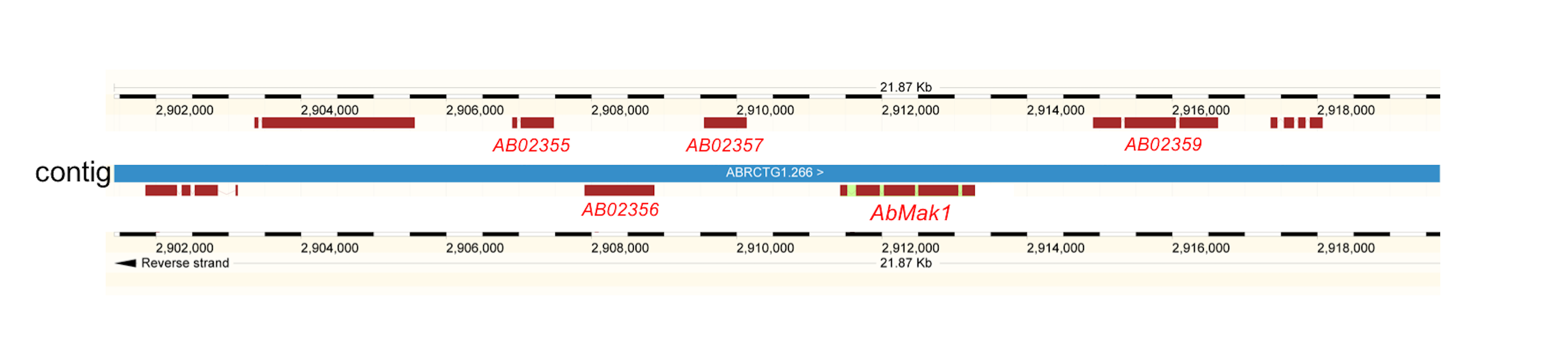

Supplement: Supplementary file 4 — Additional file 4: Figure S4. Schematic map of the genomic region including AbMak1 and flanking genes. This map was generated and modified from the Alternaria Genomes Database (http://alternaria.vbi.vt.edu/index.html). Genes are indicated as red boxes and positions of introns by white bars. [file 40694_2016_29_MOESM4_ESM.tiff]
